# Recovery, quality of life and everyday occupations among older and younger adults with severe mental illness – a comparative cross-sectional study

**DOI:** 10.1186/s12877-026-07359-0

**Published:** 2026-03-25

**Authors:** Mona Eklund, Elisabeth Argentzell

**Affiliations:** Department of Health Sciences, Lund University, Box 157, Lund, 221 00 SE Sweden

**Keywords:** Activity, Ageing, Daily occupations, Recovery, Quality of life, Well-being

## Abstract

**Background:**

Current research addressing recovery and everyday occupations among individuals with severe mental illness tends to focus on people of working age, whereas those aging with such conditions receive little attention. Whether younger and older users of mental health services differ regarding how they perceive their everyday occupations, personal recovery and quality of life seems unknown. This would be important knowledge in the support of people aging with a severe mental illness.

**Aim:**

To explore whether older and younger mental health service users differ in how they view and lead their everyday lives, focusing on everyday occupations, recovery and quality of life.

**Method:**

This was a comparative study including two subsamples. One was 49 older Day center (DC) attendees (≥ 65) and the other 65 DC attendees of working age (< 65). There was a two-year interval between data completion for the first subsample (2015) and the start of data collection for the second (2017). Data collected included measures of everyday occupations, personal recovery, and quality of life. Non-parametric statistics were used.

**Results:**

The older and the younger groups were comparable on satisfaction with everyday occupations and quality of life. The older group spent more hours in the DC and rated their occupational engagement higher, whereas they rated their activity level lower than did the younger group. There was a trend towards the older group having a higher level of recovery compared to the younger group.

**Conclusion:**

The groups were comparable in most of the areas investigated with the older group having a slight advantage. When living with a mental illness, expectations on life may be modified over the years resulting in higher acceptance of the situation. Additional studies are needed to further investigate possible difference between these two groups.

**Trial registration:**

The younger group was a subsample from a project registered with ClinicalTrial.gov. Reg. No. NCT02619318. Retrospectively registered: 20-11-2015. However, the current cross-sectional study was not based on the trial design and used only baseline data.

## Introduction

Although considerable research has focused on the recovery and everyday occupations of individuals with severe mental illness [1, 2], less attention has been directed toward those aging with such conditions. Moreover, although mental health issues emerging in older adulthood have received increasing attention [3–5], research on individuals aging with a long-term severe mental illness—and how they manage their daily lives and whether they feel they are still in a recovery process—remains limited. This gap in knowledge provides the rationale for the present study, which adopts an everyday activity perspective on recovery and quality of life. A deeper understanding of those who developed severe mental illness earlier in life and are now approaching retirement age, and how experiences of everyday occupations and recovery are related in this group, is necessary to enable relevant support to this group. Research indicates that this group has the capacity and willingness to maintain an active daily life, tailored to their circumstances [6].

Existing mental health services are largely planned for adults of working age, with a particular emphasis on participation in the labor market [7, 8]. People who approach retirement age might need support that focuses on other aspects of everyday occupations, such as alternative ways of being productive, having satisfying hobbies, and social engagements. Research has shown that older people with mental illness valued an established and enduring sense of identity, as well as coping strategies to provide continuity and reinforce that sense of identity [9]. This is in line with cores of the personal recovery process [10], which is complementary to clinical recovery and signifies that people with mental health problems lead an active and meaningful everyday life with personal growth although they may have lasting psychiatric symptoms [11]. The importance of continuously developing one’s identity as an older person with mental severe mental illness has been corroborated in other research, also identifying everyday occupations as a life area that could support one’s sense of well-being and identity [6]. This accords with research on adult people of working age with severe mental illness, showing that participation in everyday occupations and satisfaction with such occupations seem to be important for one’s personal recovery journey [10, 12]. In the current study, everyday occupation is defined broadly as personally meaningful tasks related to productivity, leisure, maintenance, and social activities. It has been suggested that distinguishing between the actual performance of occupations—such as participation in a variety of tasks—and individuals’ subjective perceptions of those occupations in terms of meaningfulness and satisfaction may yield valuable insights. Subjective experiences of everyday occupations have been found to be more closely associated with various aspects of recovery and quality of life than mere occupational performance [13–17]. That research was based on people with mental illness of all ages and, to the best of our knowledge, studies investigating whether the same would be valid for older people seem to lack.

Municipal psychiatric services, such as day centers (DC), are offered to people with severe mental illness in order to support visitors towards a meaningful and active everyday life [18] and enhance their personal recovery [19]. DCs can admit adults of all ages, also older adults with severe and complex mental illness. Comparing two groups with severe mental illness—one older and one younger—that share similarities in disease severity, everyday occupations, and type of mental health support could provide insights into potential age-based differences concerning everyday occupation and well-being in terms of personal recovery and quality of life. Choosing the DC context for such a comparative study would provide similarity in both everyday occupational situations and type of mental health support. Just like quality of life, personal recovery is regarded a vital outcome of mental health support [20], also for older people [9], and the two phenomena have been found to be closely related [21]. Identifying how an older group with severe mental illness perceive their everyday occupations, compared to younger individuals, is important considering the close links identified between satisfying everyday occupations and recovery, quality of life [12, 22] and meaning in life [23] among people with mental illness of working age. Existing research does not provide a clear picture of whether patterns of such relations differ between older people and people of all ages. Such knowledge would be valuable for informing and tailoring support for older people with severe mental illness.

Against the above backdrop of inconclusive research, this study employed an explorative approach. The aim was to explore:

- whether and how older and younger people with severe mental illness visiting DCs differ in how they view and lead their everyday lives with a focus on everyday occupations (both actual performance and subjective perceptions), personal recovery and quality of life.
- whether associations between the studied aspects of everyday occupations and estimates of personal recovery and quality of life would differ between an older and a younger group.

## Methods

This was a comparative study including subjects from two projects, both of which were approved by the Ethical Review Board in Lund (reg. nos. 2012/70 and 2013/456). The ethical guidelines stipulated in the Swedish law on research on humans [24] and the Helsinki declaration [25] were followed.

### Study context

The study was performed in the DC context in Sweden. In many Western countries, individuals with severe mental illness are offered participation in municipal DC to maintain a meaningful everyday life and daily routines [19]. DC can focus mainly on work, including workshops (woodwork, weaving, etc.), home maintenance (cooking, cleaning, gardening, etc.) and a variety of services (carwash, food catering etc.) [26]. In most Swedish municipalities, access to the DC is based upon assistance assessment by a care organizer [18]. This means that individuals who wish to attend a DC apply for that, and those considered in need of such assistance receive it. Research shows that attendees tend to identify the DC as supporting their recovery journey [27, 28], although increased user involvement has been called for [19].

### Recruitment

This study included two subsamples that followed identical recruitment and data-collection procedures, separated by a two-year interval between the start of data collection for the first subsample (2017) and data completion for the second subsample (2015). The first subsample is from a project that includes the current study and addresses everyday occupations among older mental health services users who are transitioning into retirement age [29]. Starting in 2017, this project had to be set on hold during the COVID-19 pandemic (2020–2023) and was completed in 2024. The intended age span for this subsample is 60 years and older. The second subsample is from a previously reported project evaluating the activity- and recovery-oriented program Balancing Everyday Life (BEL) [30]. A subgroup in that project were DC attendees of working age, which meant an age span between 18 and 65 years. Only baseline data was included in the current study.

Except for age, the inclusion criteria were the same for both subsamples. One criterion was receiving DC support. This also meant fulfilling the requirements for that type of support, which includes having a psychiatric disability, often defined as lasting (> 2 years) difficulties in handling everyday life [31]. Good command of Swedish was an additional criterion. Exclusion criteria were comorbidity of cognitive impairments and substance use disorder as the main diagnosis.

People who met the inclusion and exclusion criteria received written and oral information from a gatekeeper, who was an employee at the DC and served as contact person between the research team and the DC attendees in the recruitment phase. The information contained details about the study, its aims and methods, and stated the voluntariness of participation and the possibility at any time to cancel one’s participation. Participants were included consecutively based on expressed interest after the meeting or during the following two weeks. This procedure did not allow for exact calculation of participation rate, but it is estimated at 75%. Those who wished to participate gave their written consent. Participants who accepted were invited to an individual meeting with a project assistant for data collection, which took place at the respective DC. This way, 49 older attendees (≥ 65 years) and 65 DC attendees of working age (< 65 years) were recruited. Nobody had salaried employment, whether in the older or the younger group.

### Data collection

A background questionnaire addressed socio-demographic information and self-reported data on psychiatric diagnosis and perceived psychiatric problems. These self-reports were subsequently assessed by a specialized psychiatrist who assigned ICD-10 diagnoses [32] to each participant. Diagnoses were subsequently categorized into four groups based on the ICD-10: psychoses, depression and/or anxiety, Asperger’s or attention deficit disorder, and other diagnosis (such as eating disorders or personality disorders). Rating scales were used as well, as detailed below.

#### Profiles of Occupational Engagement in People with Schizophrenia (POES)

The POES instrument assesses occupational engagement and is composed of two parts where the first is a 24-hour time-use report, designed with preset one-hour time slots and four columns to fill out: the activity performed, social environment (if alone or with whom), the place where the activity was performed, and feelings/thoughts in relation to the activity situation [33, 34]. The second part is a nine-item rating scale where the respondent reflects on the diary content from different aspects that operationalize occupational engagement in the POES instrument, such as balance between activity and rest, degree of variation among one’s everyday occupations, and the degree of healthy routines reflected in the diary. A four-point rating scale from 1 (don’t agree at all) to 4 (fully agree) is used, thus resulting in a possible score between 9 and 36. Good psychometric properties have been reported for an initial version where part two is assessed by an occupational therapist [33, 34]. The self-report version used in the current study has not been extensively investigated for psychometric properties but content validity, as evidenced by approval from both occupational therapists and clients engaged in various types of activity-based psychiatric rehabilitation [30, 35], has been established. Internal consistency based on the current sample was alpha = 0.80 for the older group and 0.85 for the younger group. Occupational engagement, as measured by the POES, integrates both actual performance and the subjective experience of everyday occupations by considering not only what is done, but also how the activity is perceived by the individual.

#### Satisfaction with daily occupations

The interview-based SDO questionnaire addresses two aspects of everyday occupations – activity level and satisfaction with occupations [36]. The questionnaire has 13 items addressing four domains of everyday occupations: work (3 items), leisure (3 items), home and maintenance (4 items), and personal care (3 items). Each item is answered in two steps; first the respondent answers whether they currently perform the named occupation or not (yes/no). The activity level score, which reflects actual performance, is obtained by summarizing the number of affirmative answers and scores may thus vary between 0 and 13. Second, the respondent rates their subjective experience in terms of satisfaction with being engaged in the named occupation (if affirmative answer in the first step), or their satisfaction with not being engaged in the occupation (if negative answer in the first step). A rating scale varying from 1 (worst possible satisfaction) to 7 (best possible satisfaction) is employed, rendering scores that may vary between 13 and 91.

The activity level scale addresses a variety of occupations and corresponds to a formative model, i.e., the items are not supposed to be correlated and form a common construct. This means that properties such as factor structures and internal consistency are not relevant [37], but content validity has been established for the activity level scale [38]. Satisfactory psychometric properties in terms internal consistency and construct validity have been obtained for the satisfaction scale [36].

#### Manchester Short Assessment of Quality of Life (MANSA)

A Swedish version of the widely used MANSA instrument was employed to assess self-reported quality of life [39]. It contains 12 items, one of which asks for general life satisfaction and the others addressing satisfaction with various life domains, such as personal finances, interpersonal relations, housing, work, personal safety, etc. The response scale is a seven-point scale varying from 1 (worst possible satisfaction) to 7 (best possible satisfaction). For the current study, general life satisfaction was analyzed separately, and the 11 domain-specific items were summed into a scale, where scores could vary between 11 and 77. The MANSA has shown good psychometric properties in terms of internal consistency and construct validity [39–41].

#### Questionnaire about the process of Personal Recovery (QPR)

The QPR was used to investigate participants’ perceptions of personal recovery. It is a self-report questionnaire consisting of statements that address aspects such as self-worth, connections with others, and sense of control. Participants rate their agreement with each statement by selecting one of five options, ranging from 0 (strongly disagree) to 4 (strongly agree). The Swedish version used in the current study, the QPR-Swe, contains 16 items and has demonstrated good psychometric properties, including strong internal consistency and satisfactory construct validity [42]. The sum scores may range between 0 and 64.

### Data analysis

The instruments used formed ordinal scales and non-parametric statistics were considered appropriate for the analyses. Spearman correlations were employed to analyze relationships between variables and the Mann-Whitney test to calculate group differences regarding ordinal data. The chi square test was used for comparing groups on categorical data. Associations were classified as ‘very strong’ (ρ ≥ 0.90), ‘strong’ (0.70 > *ρ* < 0.89), ‘moderate’ (0.40 > *ρ* < 0.69), ‘weak’ (0.10 > *ρ* < 0.39), or ‘negligible’ (*ρ* < 0.10) [43]. To compare the strengths of correlation coefficients, the correlations were first subjected to Fisher z-transformation to accomplish normal distribution. Then the z-value for the difference between two correlations was calculated according to the formula: z observed = (z1 – z2) / (square root of [ (1 / N1–3) + (1 / N2–3)]). An online calculator was used for this purpose: Free Significance of the Difference between Two Correlations Calculator - Free Statistics Calculators https://www.danielsoper.com/statcalc/calculator.aspx?id=104. The *p*-value for statistical significance was set at *p* < 0.05. However, since this was an explorative study, we also set a limit for non-significance, and only *p*-values > 0.1 were regarded as being statistically non-significant. The software used was the SPSS [44].

## Results

### Characteristics of the study sample

The study sample consisted of 114 individuals, 65 of whom were of working age, and 49 belonged to the older age group. Logically in relation to the selection criteria, the older group was about 20 years older than the younger ones. The age groups did not differ regarding gender. There was a statistically significant difference between the two groups on diagnosis; the category comprising Asperger’s and attention deficit disorder was more common than expected in the younger group. The distributions on the other three categories (psychosis, depression/anxiety, other) did not differ between the groups. Nor was there a group difference regarding using psychotropic medication or not (*p* = 0.118).

Concerning educational background, there was a tendency, on the border to statistical significance, that completed high school as highest education was more common in the younger group, whereas completed college education was more common in the older group (*p* = 0.05). Another tendency concerned that the younger group more often reported having a friend (*p* < 0.1). A factor that did differ between the age groups was having children, which was more common in the older group (*p* < 0.05). Further sociodemographic characteristics investigated were civil status (married or living with a partner versus not) and having a foreign background (being born in Sweden or not), where no age group differences were found. More details are displayed in Table 1. 

**Table 1:** Sociodemographic characteristics of the participants

Characteristics	The older group (*N* = 49)	The younger group (*N* = 65)	*P*-value
Sex (% women)	60	63	Ns. (0.637)
Age (mean, SD)	64 (4)	45 (11)	< 0.001
Education (%)			0.05
Nine-year compulsory school or lower	33	28	
High school	41	61	
College/university education	26	11	
Being foreign born (%)	14	9	Ns. (0.417)
Living with a partner (%)	29	19	Ns. (0.219)
Having children (%)	74	52	0.018
Having a friend (%)	74	86	0.098
Diagnosis (%)			0.007
Psychosis	30	25	
Depression/anxiety disorder	46	40	
ADHD/Aspergers	17	27	
Other	7	9	

*P*-vales  > 0.10 are considered non-significant

### Everyday occupations, personal recovery and quality of life in the two groups

Table 2 shows the results regarding occupational factors, personal recovery and quality of life. The older group spent significantly more hours in the DC per week and rated their occupational engagement higher compared to the younger group. There was also a tendency (*p* < 0.1) that the older group scored higher than the younger on personal recovery. On the other hand, the younger group were involved in more occupations, as indicated by their higher ratings on activity level. No group differences were found regarding satisfaction with everyday occupations or quality of life. 

**Table 2:** Occupational factors, personal recovery and quality of life in the two age groups

Variable	Possible range	The older group (*N* = 49)	The younger group (*N* = 65)	*P*-value
DC participation; hrs. per week	1–40	14.6 (8.6)	10.3 (8.7)	0.005
Occupational engagement; mean (SD)	9–36	28.6 (5)	22.3 (5)	< 0.001
Satisfaction w. daily occupations; mean (SD)	13–91	68.8 (13.7)	70.6 (15.3)	Ns (0.292)
Activity level; mean (SD)	0–13	6.6 (1.7)	7.8 (1.8)	0.001
Personal recovery; mean (SD)	0–64	42.9 (10)	38.6 (11)	0.084
Quality of life; mean (SD)	11–55	38.2 (8.6)	35.6 (9.2)	Ns (0.203)

### Associations between occupational factors, recovery and quality of life in the two groups

Correlations between the occupational factors (occupational engagement, activity level, and satisfaction with everyday occupations) on the one hand and personal recovery and quality of life on the other are displayed in Table 3. A similar pattern appeared for the two groups regarding statistically significant and non-significant associations. The correlations between occupational engagement and satisfaction with everyday occupations on the one hand and personal recovery and quality of life on the other were statistically significant. Regarding activity level, here framed as actual doing, all correlations to the well-being variables were weak or negligible, although statistically significant in one case—activity level was related to personal recovery in the older group. Also shown in Table 3, the test revealing whether correlation coefficient strengths differed between the two groups resulted in non-significant z scores. 

**Table 3:** Associations between the occupational factors and the respective correlates of personal recovery and quality of life in the two age groups

	The older group (*N* = 49)	The younger group (*N* = 63)	Test for *r*^2^ difference ^1)^
	Personal recovery		
Occupational engagement	r^2^=0.67, *p* < 0.001	r^2^=0.47, *p* < 0.001	z = 1.514, *p* = 0.130
Satisfaction with everyday occupations	r^2^=0.53, *p* < 0.001	r^2^=0.33, *p* = 0.008	z = 1.271, *p* = 0.204
Activity level	r^2^=0.33, *p* = 0.022	r^2^=0.12, *p* = 0.356	N.A.
	Quality of life		
Occupational engagement	r^2^=0.39, *p* = 0.006	r^2^=0.59, *p* < 0.001	z = 1.338, *p* = 0.180
Satisfaction with everyday occupations	r^2^=0.64, *p* < 0.001	r^2^=0.46, *p* < 0.001	z = 1.341, *p* = 0.180
Activity level	r^2^=0.10, *p* = 0.497	r^2^=0.23, *p* = 0.067	N.A.

^1)^ The test for difference in strength was not applicable (N.A.) for non-significant correlations

## Discussion

The findings indicate that, in most cases, the older group was neither advantaged nor disadvantaged compared to the younger group. No group differences were identified regarding satisfaction with everyday occupations or quality of life. Where a difference was found, the older group had often a more beneficial situation. This concerned time spent in the DC and the participants’ level of occupational engagement. There was also a tendency, although not statistically significant, for the older group to perceive better personal recovery. This may be related to what previous research addressing satisfaction factors has found, that when living with compromised health, be it physical or mental, people tend to adjust to their situation over time and find ways to adjust and maintain a positive outlook on their health and well-being [45].​ This has been termed response shift, which may take place because respondents change their internal standards, change their values, or redefine the outcome in question [46, 47]. Thus, when living with a mental illness, expectations on life may be modified over the years and what in younger age would have been perceived as less satisfactory may in older age be accepted as fair.

That the older group spent more hours in the DC and rated their occupational engagement higher might be two sides of the same coin. Nevertheless, the finding is not totally expected. It is known from previous research that younger DC visitors are more engaged in society at large, and are motivated to go to concerts, sports events, etc [48]. The fact that the younger group rated their activity level higher than the older group in the current study aligns with that prior finding. Intuitively, taking part in mainstream events would be perceived as being highly engaged, compared to visiting a DC. Again, however, a response shift may have occurred, such that the older group had adjusted their expectations to being a DC attendee where they felt engaged and did things that matched their capacities, whereas the younger group wanted to fit into society at large without always feeling at ease and fully engaged there. This is speculation, however, which should be explored in qualitative research, but according to the measure used (POES) in the current study, occupational engagement includes not only performing occupations but also social and geographical diversity [33], which may be harder to accomplish on one’s own compared to the helping atmosphere in a DC [28].

There was a trend (*p* < 0.10) suggesting that the older group reported higher levels of personal recovery than the older group. This is interesting, since the older group had had their mental for several years, but is in line with prior research showing that older people with mental illness experience personal recovery [9]. The fact that older persons tended to have a higher level of recovery points to a possible benefit of organizing the DC programs in such a way that the older attendees could support the recovery journey of the younger ones, in the shape of informal or formal peer support. Formal peer support has been shown to promote [49], and even be a cornerstone [50] in recovery.

When everyday occupations are viewed from different perspectives—such as actual doing, subjective experience, or a combination of both [13]—activity level reflects actual doing. Occupational engagement represents a blend of actual doing and subjective experience, while satisfaction with everyday occupations is considered a purely subjective experience. Applying this reasoning on the current findings, it seems logical that the result patterns for occupational engagement and satisfaction with everyday occupations differ from that for activity level.

How subjectively perceived occupational factors are related to well-being in terms of personal recovery and quality of life among older persons with severe mental illness has not been investigated before, but a study performed in the context of supported housing, where some residents had reached retirement age, found that such relationships seemed weaker and fewer compared to existing research on younger samples [12]. When comparing the strength of associations in the current subsamples, no statistically significant differences were found, however, giving no reason to assume that such well verified relationships between subjectively perceived everyday occupations and well-being [51–53] would vary by age. Just as in prior research, quality of life and personal recovery were moderately related with occupational engagement in both sub-samples.

Taken together, the results suggest that both groups were similar in their satisfaction with everyday occupations and quality of life. This indicates that they require comparable rehabilitation support—something that does not appear to be the case in practice and seems to disadvantage the older target group [6]. The trend of older adults showing greater recovery suggests that they could serve as a valuable source of support for the younger group in their personal recovery journeys.

### Methodological considerations

Although the two samples were drawn from separate studies, they were comparable across most known parameters, aside from differences attributable to age. The age difference probably explains why a larger proportion of the older group had children and had completed a higher education. Furthermore, procedures for recruitment and data collection were the same for both samples and all instruments have proven to be psychometrically sound. These facts strengthen the internal validity of the study. The samples used would also be representative of the target population of people with mental illness visiting DCs. Yet, the subsamples included were quite small, and it is possible that type-II errors have occurred and that differences, such as the trend concerning personal recovery, have been underestimated from a statistical point of view. The small sub-samples also limit the external validity of the study, and more research into everyday occupations and well-being among people ageing with mental illness is warranted to replicate and thereby validate the current findings of everyday occupations.

Another factor that may be discussed is the potential occurrence of a history effect, as the two data sets were not collected simultaneously. However, we consider this risk to be minimal, as only a few years separate the two data collections. Data for the younger subsample was collected before the COVID-19 pandemic. In contrast, data collection for the older group began before the pandemic but was paused for an extended period until all restrictions were lifted and COVID-19 was considered a seasonal flu. Another potential flaw is that the POES self-report version used in this study has not been extensively psychometrically tested, although its homogeneity and face validity have been shown, both in the current project and in previous research [30, 35].

## Conclusion

The older and the younger groups were comparable regarding satisfaction with everyday occupations and quality of life. The few differences identified indicate that the older group seemed slightly advantaged compared to the younger group of DC attendees. They spent more hours in the DC and rated their occupational engagement higher, whereas they rated their activity level lower than did the younger group. There was also a tendency towards higher personal recovery ratings in the older group. Possible explanations to these findings might be that a response shift takes place alongside ageing, that people adjust their expectations and upgrade their satisfaction although actual life circumstances may not have improved. Whether and how a response shift actually takes place in relation to phenomena such as occupational engagement, satisfaction and meaning is something that should be investigated in future research.

## Data Availability

The data sets analysed for the current paper are not publicly available due to the restriction set by the Swedish [Act concerning the Ethical Review of Research Involving Humans](http:/codex.vr.se/en/manniska5.shtml)but are available from the corresponding author on reasonable request.

## Abbreviations

BEL: Balancing Everyday Life
DC: Day Center
ICD: International classification of diseases
MANSA: Manchester Short Assessment of Quality of Life
POES: Profiles of Occupational Engagement among people with Severe mental illness
QPR: Questionnaire about the process of Personal Recovery
SD: Standard deviation
SDO: Satisfaction with daily occupations
